# Copy number variation signature to predict human ancestry

**DOI:** 10.1186/1471-2105-13-336

**Published:** 2012-12-27

**Authors:** Melissa Pronold, Marzieh Vali, Roger Pique-Regi, Shahab Asgharzadeh

**Affiliations:** 1 Department of Pediatrics, Children’s Hospital Los Angeles and The Saban Research Institute, Keck School of Medicine, University of Southern California, Los Angeles, CA, USA; 2 Department of Preventive Medicine, Keck School of Medicine, University of Southern California, Los Angeles, CA, USA; 3 Department of Clinical and Translational Science, School of Medicine, Wayne State University, Detroit, MI, USA

## Abstract

**Background:**

Copy number variations (CNVs) are genomic structural variants that are found in healthy populations and have been observed to be associated with disease susceptibility. Existing methods for CNV detection are often performed on a sample-by-sample basis, which is not ideal for large datasets where common CNVs must be estimated by comparing the frequency of CNVs in the individual samples. Here we describe a simple and novel approach to locate genome-wide CNVs common to a specific population, using human ancestry as the phenotype.

**Results:**

We utilized our previously published Genome Alteration Detection Analysis (GADA) algorithm to identify common ancestry CNVs (caCNVs) and built a caCNV model to predict population structure. We identified a 73 caCNV signature using a training set of 225 healthy individuals from European, Asian, and African ancestry. The signature was validated on an independent test set of 300 individuals with similar ancestral background. The error rate in predicting ancestry in this test set was 2% using the 73 caCNV signature. Among the caCNVs identified, several were previously confirmed experimentally to vary by ancestry. Our signature also contains a caCNV region with a single microRNA (*MIR270*), which represents the first reported variation of microRNA by ancestry.

**Conclusions:**

We developed a new methodology to identify common CNVs and demonstrated its performance by building a caCNV signature to predict human ancestry with high accuracy. The utility of our approach could be extended to large case–control studies to identify CNV signatures for other phenotypes such as disease susceptibility and drug response.

## Background

Copy number variations (CNVs) are gains and losses of genetic material in the human genome that are greater than 50 base pairs (bp) in size [1]. These structural variants are present in both healthy and diseased populations, and may confer susceptibility to certain illnesses through a gene dosage effect [2]. The frequency of CNVs varies by ethnicity, which may contribute to phenotypic variations and differences in disease susceptibility across different ethnic groups [3,4]. An array-based comparative genome hybridization (aCGH) performed on pooled genomic DNA from the International HapMap Project populations revealed 26 European population-specific CNVs, 53 African population-specific CNVs, and 23 Asian population-specific CNVs [5]. Several technological approaches are used to examine CNVs in the human genome. Comparative genomic hybridization techniques utilize thousands of probes to detect CNVs at a low resolution; single nucleotide polymorphism (SNP) microarray platforms employ millions of probes to detect smaller CNVs at precise locations in the genome; and the most comprehensive assessment of CNVs can be performed using next-generation sequencing of the human genome [6-8]. Numerous algorithms have been developed for array based CNV detection using the probe signal intensity from these array-based assays [9,10]. The underlying assumption is that there are two copies of each autosomal chromosome in the human genome, and the goal of these algorithms is to estimate the size and location of regions which are significantly different from this assumption.

The statistical approaches implemented for the detection of CNVs are often performed on a sample-by-sample basis. A Hidden Markov Model and Bayesian analysis are statistical approaches commonly used for single-sample CNV calling [11-18]. We previously developed the Genome Alteration Detection Analysis (GADA) algorithm to identify CNVs on aCGH and SNP microarray platforms [19,20]. GADA utilizes a Sparse Bayesian Learning (SBL) technique to determine the possible CNV locations, and then a backward elimination (BE) procedure is used to rank the CNVs for manual adjustment of the false discovery rate. The high accuracy and computational efficiency of GADA has proven its utility in very large data sets used to identify global variation in copy number in the human genome [21].

Here we demonstrate a novel and simple method to detect common CNVs, which can then be used to build a common ancestry (caCNV) signature that is predictive of ancestry. Our method uses a simple application of the GADA algorithm on a distribution of t-statistics obtained by comparing microarray probe signal intensity data of two different ancestral groups. The t-statistics arranged by the genomic locations of the probes allow detection of common genome-wide CNVs. Next the copy number state of each individual is assessed for the caCNVs and used as features in a linear discriminant analysis model to identify a caCNV signature that can predict ancestry. Lastly, we validated this CNV signature in an independent dataset of samples with similar ancestry.

## Methods

### Study populations

Individuals of European, African, and Han Chinese ancestry were available from the International HapMap Project [22]. Genome-Wide Human SNP Array 6.0 (Affy SNP 6.0) data for the HapMap individuals was obtained from Affymetrix (http://www.affymetrix.com/index.affx). The training set includes 60 unrelated HapMap individuals of European descent from Utah (CEU), 60 unrelated HapMap individuals of the African Yoruba from Nigeria (YRI), and 45 unrelated Han Chinese HapMap individuals from Beijing, China (CHB).

The test set was obtained through the Cancer Genetic Markers of Susceptibility (CGEMS) project [23]. The CGEMS dataset was available from the National Institute of General Medical Sciences (NIGMS) Human Genetic Cell Repository through dbGaP (accession: phs000211.v1.p1). This is a population-based Affy SNP 6.0 dataset of 300 samples (100 Caucasian, 100 African-American, and 100 Han Chinese) collected by the NIGMS to use as normal healthy controls (http://ccr.coriell.org/Sections/Collections/NIGMS/?SsId=8). The ethnicities for the African-American and Caucasian populations were self-identified as reported in physician records. The inclusion criteria for the Han Chinese cohort, obtained from subjects living in the Los Angeles area, were that all four grandparents were born in Taiwan, China, or Hong Kong.

### DNA microarray

The Affy SNP 6.0 consists of 906,600 polymorphic probes for detection of SNPs and CNVs, and 946,000 non-polymorphic probes for identification of CNVs only. The average minor allele frequency of SNPs on this platform in the HapMap CEU, CHB, and YRI populations is 19.5%, 18.2%, and 20.6%, respectively. CNV probes were originally selected for their genomic spacing (744,000, 79%) and based on known CNVs identified in the Database of Genomic Variants (202,000, 21%). The median distance between all SNP and CNV probes combined is < 700 base pairs [24].

### Statistical analysis

#### DNA microarray normalization and summarization

Affy SNP 6.0 data were normalized according to the manufacturer’s guidelines and using Genotyping Console 3.0 (Affymetrix Inc., Santa Clara, CA). Quantile normalization, which corrects for fragment-size amplification and GC content, was performed on data from the training and test sets using the 270 HapMap samples processed at Affymetrix, Inc. as the reference group [25]. The result is a log2ratio, which is the logarithm of the signal intensity of the probe relative to the reference value. For each polymorphic SNP probe, the log2ratio of the two alleles are summarized to produce a single log2ratio value; and one log2ratio value is estimated for each individual non-polymorphic CNV probe. The entire dataset was imported into R version 2.9.1 (http://www.r-project.org/). All the analyses were carried out in R and using R-GADA package [26].

### Identification of common CNVs using genome-wide T-statistics and GADA

The underlying assumption for human DNA copy number is that there are two autosomal copies of each chromosome, with an infrequent occurrence of nonrandom copy number gain and copy number loss throughout the genome. Therefore, under the null hypothesis that most DNA sequences consist of 2 copies, the probe signal intensities will follow an approximately normal distribution, with increases in probe signal intensity corresponding with copy number gains; and decreases with a corresponding copy number loss:

(1) $$y_{\mathrm{ij}}=x_{\mathrm{ij}}+e_{\mathrm{ij}}$$

where *y*_*ij*_ is the signal intensity of sample *i* and probe *j*.

Because the normalization step corrects for experimental bias in probe signal intensities, the number of probes spanning a CNV will share a common mean log2ratio *x*_*ij*_ corresponding to the underlying DNA copy number value. The noise *e*_*ij*_ is assumed to be zero-mean, and Gaussian.

The t-test can be used to assess whether the mean measurements of two groups are statistically different from each other. Here we use the t-test to determine whether the mean log2ratio in one population (*A*) is statistically different from the mean log2ratio in a second population (*B*).

(2) $$t_{j}=\frac{{\bar{y}}_{j}^{A}-{\bar{y}}_{j}^{B}}{\sqrt{S_{j}^{2}\left(\frac{1}{N_{A}}+\frac{1}{N_{B}}\right)}}$$

(3) $$S^{i^{2}}=\frac{\sum_{i\in A}{\left(y_{\mathrm{ij}}-{\bar{y}}_{i}^{A}\right)}^{2}+\sum_{i\in B}{\left(y_{\mathrm{ij}}-{\bar{y}}_{i}^{B}\right)}^{2}}{N_{A}+N_{B}-2}$$

(4) $${\bar{y}}_{j}^{A}=\frac{1}{N_{A}}\sum_{i\in A}y_{\mathrm{ij}}\;{\bar{y}}_{j}^{B}=\frac{1}{N_{B}}\sum_{i\in B}y_{\mathrm{ij}}$$

Pair-wise comparisons of the microarray probe signal intensity data in CEU versus YRI, CEU versus CHB, and YRI versus CHB were performed using the t-test. This approach generated *t*_*j*_ (t-statistics) for each of the 1.8 million probes on the Affy SNP 6.0. Under the null hypothesis that two human populations will have most DNA sequences in common, the t-statistics will asymptotically follow a normal distribution. The t-statistic will approximate zero for the two populations who share similar diploid genomes. A region with positive t-statistic scores would then correlate with a region showing evidence of copy number gain for one population, with the second population having either neutral or a loss of copy number for that region. Conversely, regions with negative t-statistic scores will identify regions of the genome in which copy number loss is present in one population, and is absent or contains a copy number gain in the second population. To identify regions with positive or negative t-statistics, *t*_*j*_ for the 1.8 million SNP and CNV probes are arranged based on the chromosome location and imported into GADA.

The ordered t-statistics data were used to identify significant genomic boundaries of positive or negative *t*_*j*_ values. These regions correspond to regions with discriminative copy number variations. The number of probes spanning a CNV region common to a population is assumed to share a common t-statistic value. Therefore, the objective of GADA is to identify the genome-wide CNVs which are most likely to be shared in one population, that also differ in another population. This is a simple modification of the GADA method in which t-statistics are used in place of the log2ratios. The GADA method consists of two main steps. The first step is a Bayesian learning process which generates a set of candidate breakpoints and segment means while trying to achieve an optimal balance between model fit (measured as residual sum of squares) and model sparseness (the number of breakpoints). The Bayesian learning process is driven by a prior parameter, which is determined by the amount of segmentation expected in the sample. Following the initial segmentation process, the significance of each segment is estimated as a function of the segment mean and variance. The second step is then a backward elimination procedure which removes segments with a level of significance less than the user-predefined threshold. The prior parameter (alpha) was set to a = 0.5 and the significance threshold (T) was set to T = 9 for identification of breakpoints. These estimates for the alpha and T were selected based on copy number analysis of Affymetrix SNP 6.0 array data previously described and provided the most parsimonious model [19]. Further, only significant segments with greater than 10 probes were selected for the analysis to decrease the potential for false positive results.

### Building the caCNV signature

For each *CNV*_*k*_ segment identified by GADA using the t-statistic data, the sum of the log2ratio values of the total number of probes spanning the *k*-th CNV was calculated for each individual in the training set. Thus each person was assigned a vector of features, and for the *k*-th CNV and the *i*-th individual:

(5) $$f_{\mathrm{ik}}=\sum_{k\in \mathrm{CN}V_{k}}y_{\mathrm{ij}}$$

We then used a variation of the linear discriminant analysis (LDA) approach, named nearest shrunken centroids, to identify which of these CNV features are caCNVs that can accurately be used to predict the ancestry of two defined populations. Briefly, the method computes a standardized centroid for each class, and then a weighted discriminant is computed to assess if each sample leans towards one population or the other. The shrunken centroid method has been implemented as an R package (prediction analysis for microarrays, PAMR) and used for this analysis [27]. Finally, a ten-fold cross validation was performed on the training set to estimate the performance of the model. The t-statistics were calculated and CNV models were identified during each iteration of the cross-validation routine, without splitting the parent-offspring trios.

### Validation

Validation of the caCNV signature was performed using the independent CGEMS test set, with the log2ratio sum for each sample calculated using the Affy SNP 6.0 probes spanning the caCNV derived from the training set. The self-reported ethnicities of the test set were compared to a principal component analysis (PCA) of genome-wide SNP data using a panel of 4,326 SNPs previously published as ancestry informative markers (AIMs) for African Americans [28]. ADMIXTURE version 1.21 software was used to estimate ancestry using a model-based approach from the same panel of SNPs [29]. 

## Results

### Identification of caCNVs

**Figure 1:**
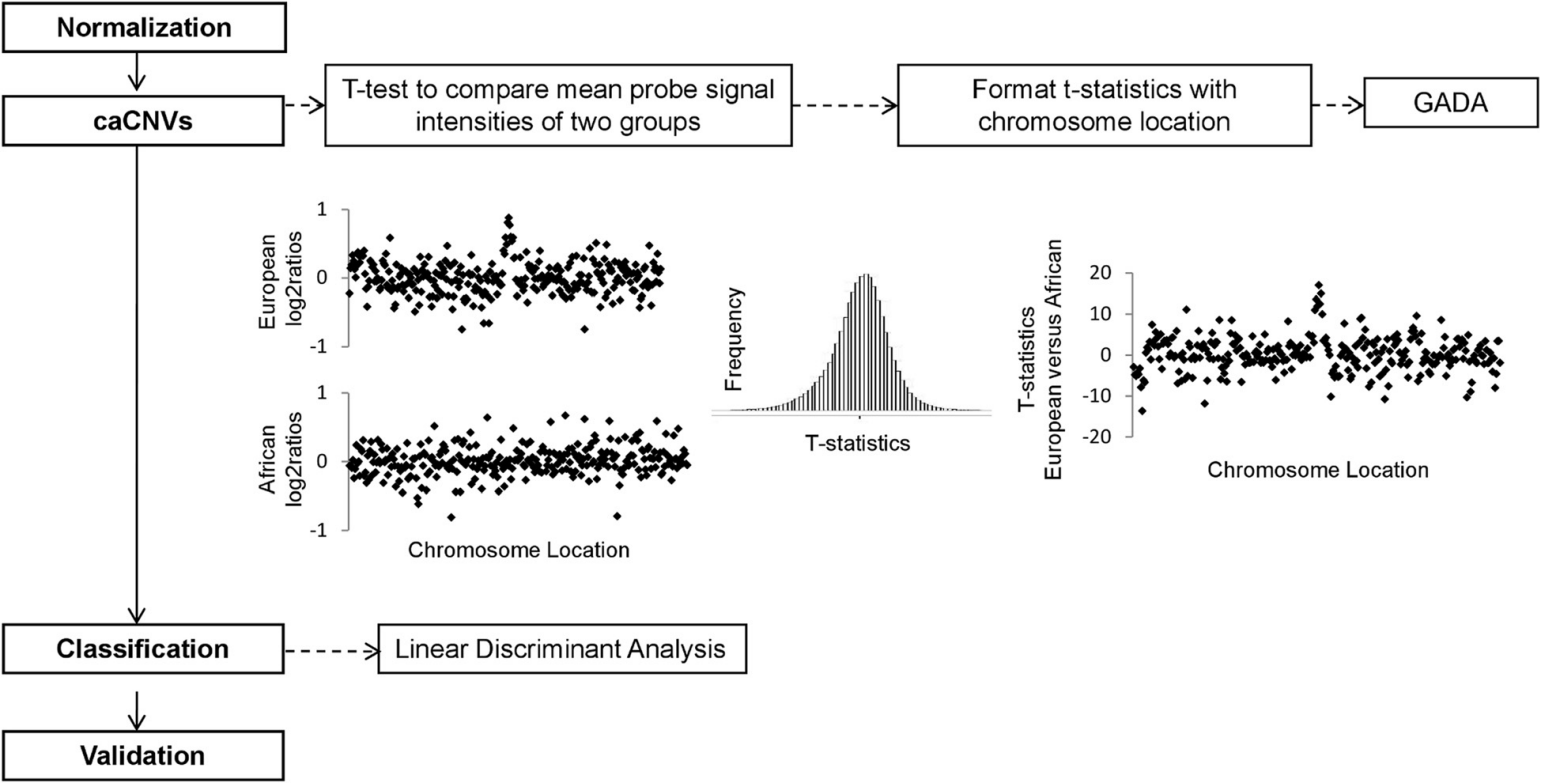
**Overview of Method.** Affymetrix SNP6.0 probe signal intensity data are normalized and summarized for copy number analysis. The mean log2ratio of each probe is compared between two populations of different ancestries using the t-test. The resulting t-statistic for each probe is formatted with chromosome position and imported into GADA to identify common ancestry CNVs (caCNVs). The t-statistics follow a normal distribution, with the t-statistic values in the tails representing the common ancestry probes. Finally, the sum of the log2ratios for each CNV is calculated and used as features in linear discriminant analysis to identify a minimum set of caCNVs required to classify the populations.

**Figure 2:**
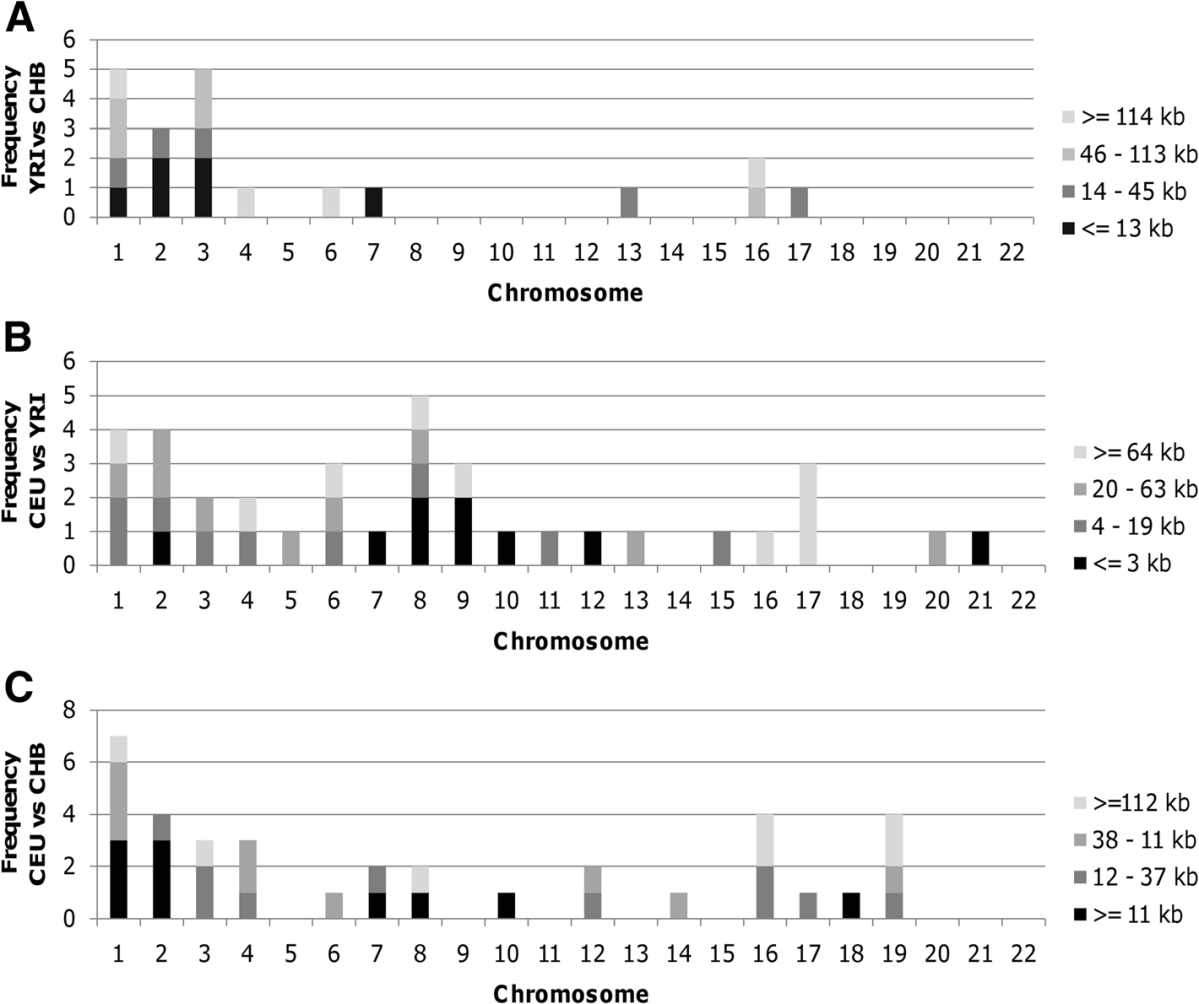
**GADA Identifies Common Ancestry CNVs Between Populations of Different Ancestry.** GADA identifies common ancestry CNVs (caCNVs) in pair-wise analysis of the three training sets (CEU, YRI, and CHB). The distribution of the caCNVs are shown for the **A**) 36 caCNVs which differ between the CEU and YRI populations (median caCNV size of 107 KB), **B**) 36caCNVs which differ between the CEU and CHB populations (median caCNV size of 73 KB), and **C**) 20 caCNVs which differ between the African and Han Chinese populations (median caCNV size of 140 kb). The frequency of caCNVs is plotted by chromosome and the color of the bar indicates the size of the caCNV.

**Figure 3:**
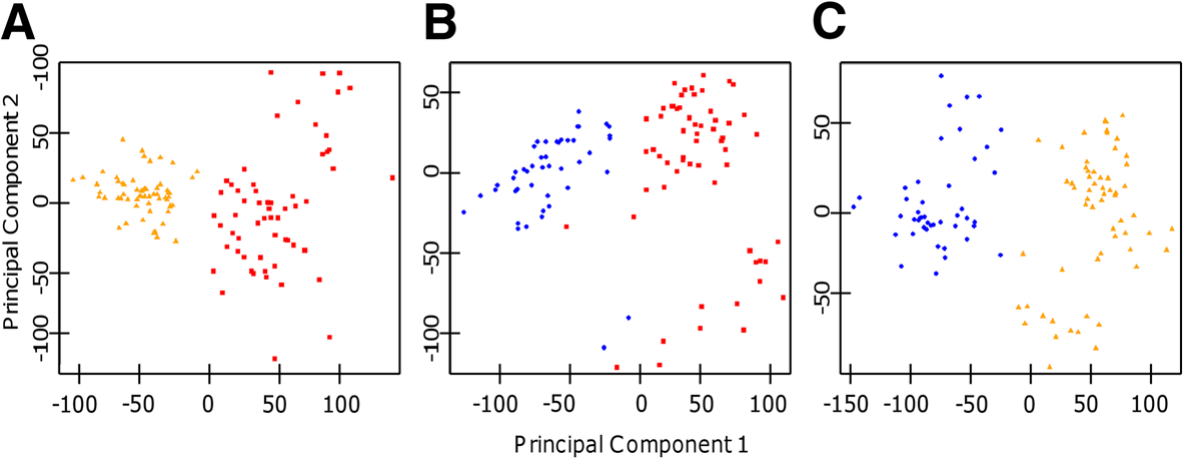
**Principal Component Analysis** (**PCA**) **using caCNVs Clusters Samples by Ancestry.** For each individual, the sum of log2ratios of the caCNVs identified using pair-wise analyses were calculated and used for PCA analyses. Scatter plots of the first two principal components of **A**) the 36 caCNVs comparing CEU versus YRI populations, **B**) the 36 caCNVs comparing CEU versus CHB populations, and **C**) the 20 caCNVs comparing YRI versus CHB populations shows good separation of individuals based on ancestry (red squares: CEU; yellow triangle: YRI; blue circle: CHB).

**Figure 4:**
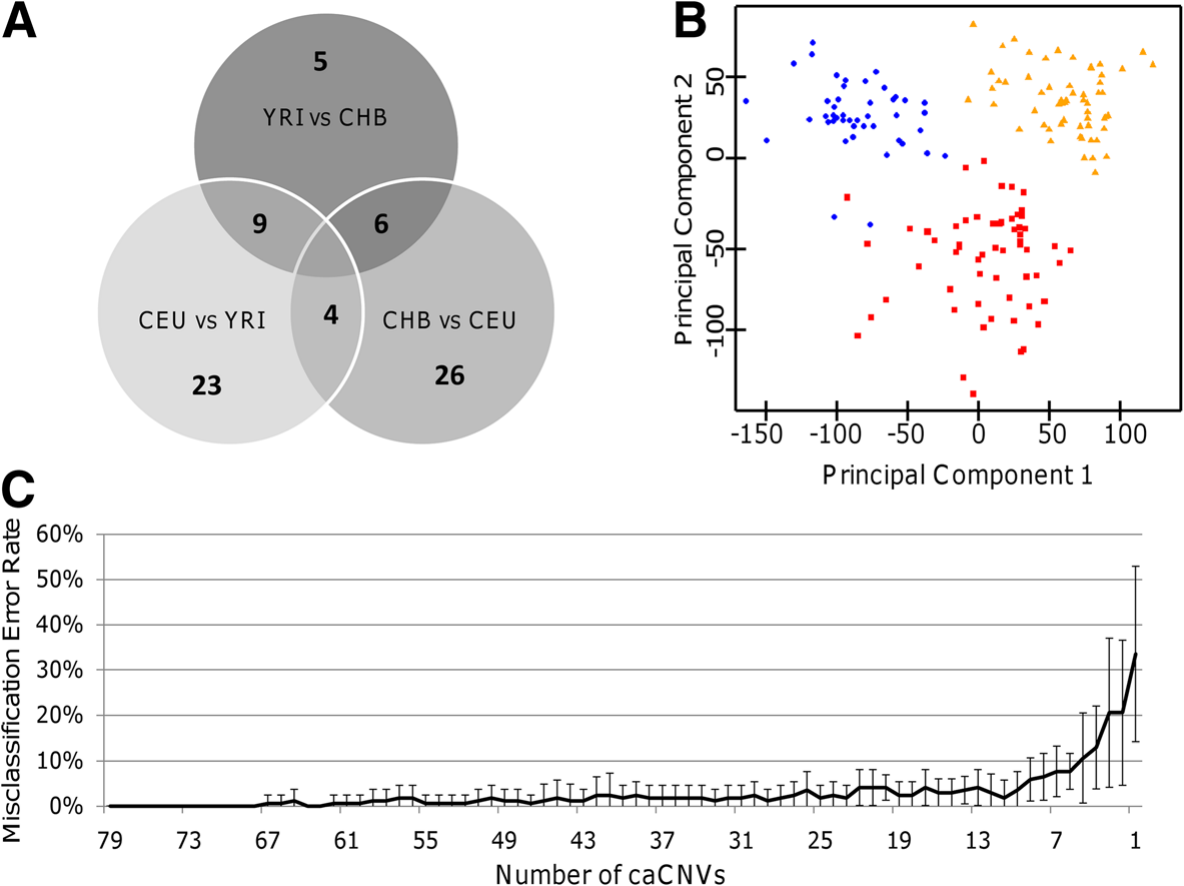
**Identification of unique caCNVs among European**, **African**, **and Han Chinese Populations. A**) Venn diagram of the 92 caCNVs identified from the pair-wise population comparisons identifies 73 unique caCNVs. **B**) Scatter plot of the top two principal components using data generated from the 73 unique caCNVs shows good separation of individuals based on ancestry (red square: CEU; yellow triangle: YRI; blue circle: CHB). **C**) Plot of the misclassification error rate for predicting ancestry using decreasing numbers of the caCNVs identified using ten-fold cross validation analyses of the training set.

**Figure 5:**
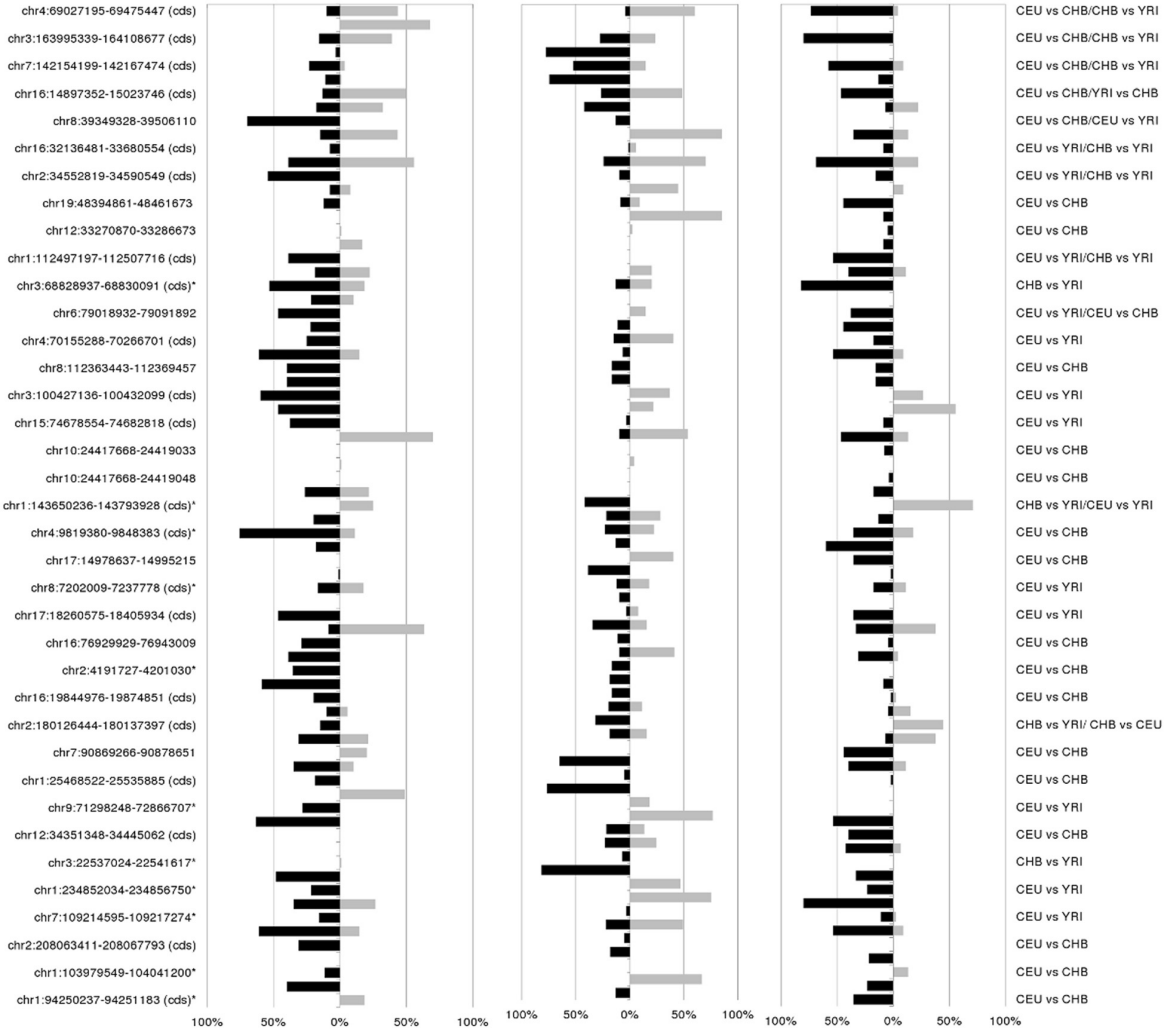
**Frequency of Copy Number Gains and Losses for the 73 unique caCNVs among the HapMap Training Set of A)European ancestry (CEU),B) African ancestry (YRI), andC) Han Chinese ancestry (CHB).** The panel is shown in ascending order (top to bottom) by statistical significance obtained using the nearest shrunken centroid analysis. The genomic coordinates of the caCNVs are based on NCBI Build 36, UCSC Version hg18. The caCNVs that affect coding sequence are designated with (*cds*) following the genomic coordinates. Those caCNVs that are novel to this analysis are indicated with an asterisk after the genomic coordinates. (copy number losses: black; copy number gains: grey).

In order to identify common CNVs that differ between two populations, a series of t-tests were performed on the mean log2ratio for each Affy SNP 6.0 probe comparing CEU, YRI, and CHB populations (Figure 1). GADA analysis of the t-statistic values of each pair-wise analysis, ordered based on the genomic location of its corresponding probe, identified 26, 31, and 16 caCNVs, respectively, which differed between training set populations of CEU and YRI ancestry, CEU and CHB ancestry, and CHB and YRI ancestry (Figure 2). A PCA of the caCNV values for each individual in the pair-wise comparisons verified the separation of these three populations (Figure 3). Of the 73 total caCNVs identified by the three pair-wise comparisons, 10 caCNVs were common in analyses comparing the YRI to the CEU or CHB populations, and 5 caCNVs were common comparing CEU or CHB against the other two populations, resulting in 73 unique caCNVs in the signature (Figure 4A). Scatter plots of the top two principal components in the PCA of the 73 caCNV values generated for each individual in the training set verified the separation of these three populations (Figure 4B). The median genomic size of the caCNV signature was 29.3 kilobases (range 1.4 – 1544.1 kilobases). The caCNVs were located on all autosomal chromosomes except for chromosomes 21 and 22. Figure 5 shows the distribution of copy number gains and losses of the 73 caCNVs across individuals of the three ancestral groups. Among the caCNVs, losses were more commonly observed across the three populations. The individual CNVs detected for each sample are listed in Additional file 1: Table S1.

### CaCNV signature-based ancestry classification

Nearest shrunken centroid analysis using the 73 caCNV signature in the training set separated the CEU, YRI, and CHB populations with 1.7% error using the ten-fold cross-validation routine (Figure 4C, Additional file 2: Table S2). As few as 25 caCNVs could be used to predict ancestry with less than 10% error. The most significant caCNV was located in chromosome 4q13.2, with 43%, 60%, and 4% of the CEU, YRI, and CHB populations exhibiting copy number gains; and 10%, 4%, and 73% with copy number losses. This region encompassing the UDP-glucuronosyltransferase 2B17 (*UGT2B17*) gene has previously been reported to be deleted in East Asian populations by DNA sequencing [30-32]. The second most significant caCNV was located on chromosome 3q26.1 and contains only a microRNA (*MIR720*). The third most significant caCNV is a duplicated region of chromosome 17q21.31 found only in Europeans, which has been validated experimentally by fluorescence in situ hybridization (FISH) and next-generation sequencing techniques [31,33].

### Independent validation of the caCNV signature

**Figure 6:**
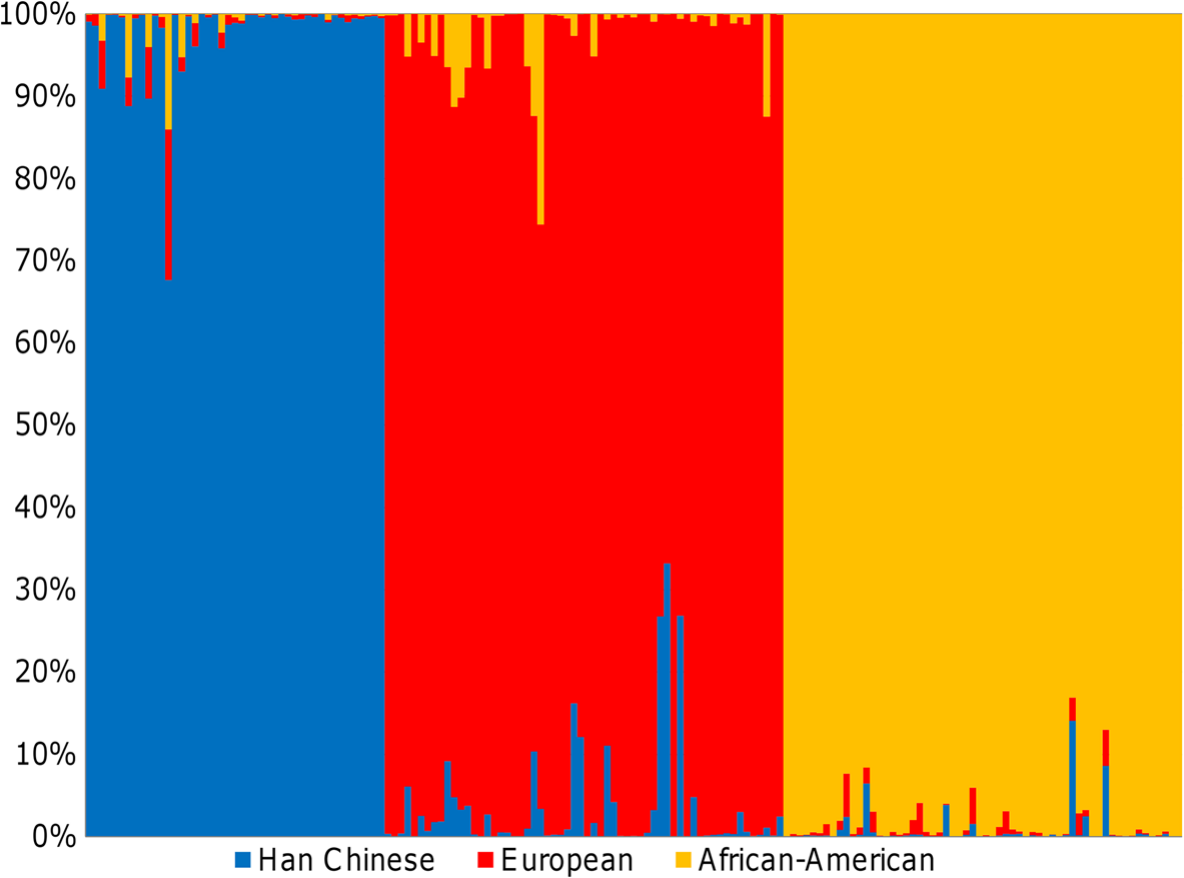
**Estimated Probability of Ancestry Classification using caCNV Signature.** The 100 European, 100 African-American, and 100 Han Chinese test samples are plotted against the estimated probability of belonging to each population. Each vertical bar represents an individual. The height of each bar is proportional to the probability that the individual belongs to a given ancestry (red bars: European; yellow bars: African-American; blue bars: Han Chinese).

The entire test set of 100 Han Chinese samples, 98 out of the 100 African-American samples, and 96 out of the 100 European samples were correctly classified using the 73 caCNV signature, with overall misclassification error rate of 2% (Figure 6). PCA was performed on a panel of 4,326 genome-wide SNPs used as AIMs to verify the separation of these three populations by self-reported ancestry (Additional file 3: Figure S1A) [28]. To further investigate the effects of admixture on classification, ADMIXTURE version 1.21 software was used to estimate ancestry using a model-based approach from the same AIMs panel of 4,326 SNPs [29]. The estimates of ancestry for each individual using the caCNV signature and genome-wide SNPs were correlated in the Han Chinese (R^2^ = 0.974), Europeans (R^2^ = 0.924), and African-Americans (R^2^ = 0.914), confirming the accuracy of the caCNV signature (Additional file 3: Figure S1).

## Discussion

This study shows a novel methodology for identifying a common CNV signature that could predict ancestry with an extremely high accuracy. Our 73 caCNV signature distinguishes European, African, and Han Chinese ancestry with an error rate of only 2%. In our signature, we also report the identification of the first microRNA caCNV. Importantly, our approach is applicable to a wide range of biomedical research aimed at identifying CNV signatures predictive of population phenotypes.

Existing methods for CNV detection are often performed on a sample-by-sample basis, which is not ideal for large datasets where common CNVs must be estimated by comparing the CNVs of the individual samples. Our proposed method identifies population-level CNVs using an application of our published GADA method. Common CNVs are determined directly from the t-statistics estimated by comparing the microarray probe signal intensities of populations of different ancestry. When used in a linear discriminant analysis model, a subset of 73 CNVs could accurately predict population structure. The average Vst for the caCNVs identified in our study was 0.31 (range 0.04 - 0.82). Vst calculations range from 0 (no population differentiation) to 1 (complete population differentiation). The distribution of CNVs in the human genome has previously been shown to vary by ethnic populations [34]. In total, 53/73 (73%) caCNVs discovered using our method has previously been identified as population differentiated. Our method identified 52 caCNVs which overlapped with a human CNV map previously developed using the same HapMap populations [35]. Many of the caCNVs we discovered have previously been validated experimentally (Additional file 2: Table S2). For instance, 14 of the caCNVs in our signature were previously reported as population differentiated using the HapMap samples using multiplex ligation-dependent probe amplification [5]. We have also identified caCNVs on chromosomes 4q13.2, 16p13.11, 17p11.2, 17q12 and 17q21.31 that have been confirmed by DNA sequencing [31]. In addition to sequencing, the caCNV on chromosome 17q21.31 has been validated using FISH analysis and the caCNV on chromosome 17q12 was validated using quantitative PCR [33]. The most significant caCNV we identified in our analysis was on chromosome 4q13.2. This region encompassing the *UGT 2B17* gene shows exceptionally increased population variation, and is most frequently deleted in East Asian populations [30-32]. Another significant caCNV in our analysis was in the region of chromosome 3q26.1. W report a copy number loss in 80% of the Han Chinese population, which is consistent with previous reports [5,34]. This region contains only a microRNA (*MIR720*) that has been shown to be expressed in melanocytes and melanoma [36]. Finally, we demonstrate novel caCNVs located throughout the genome on chromosomes 3p24.3, 3q12.1, 3q13.12, 4p16.1,7q31.1, 8p23.1, and 14q32.33.

Our approach in building a common CNV signature has several advantages. First, the proposed t-test approach is a quick and simple method to identify regions of DNA copy number which are significantly different in two populations. The GADA prior parameters provide users the flexibility to control sensitivity and specificity in identifying boundaries of common CNVs on the t-statistic data. These adjustments can be made in real-time as only one dataset (t-statistic values) is analyzed in GADA. In comparison, the Significance Testing for Aberrant Copy Number (STAC) algorithm creates a binary matrix from the normalized microarray probe signal intensities of individual samples, assigning genomic regions with no copy number change to zero and genomic regions with copy number gains or losses to one. Regions of copy number variation are then determined by their length and frequency of occurrence. STAC uses non-overlapping windows to search for evidence of CNVs in each chromosome, which can be computationally expensive when using small window sizes. Mei et al. ran the STAC algorithm longer than 48 hours on a 3 GHz windows PC with 4 Gb of RAM to analyze >32,780 non-overlapping windows of chromosomes 1–22 of 112 HapMap samples [37]. While GADA provides significant speed and flexibility in controlling for false discovery rate, the breakpoint detection analysis of the t-statistics values could be accomplished using other approaches such as Circularly Binary Segmentation. Another advantage of our approach is the elimination of data reduction techniques such as principal component analyses to identify common CNVs or the use of principal component values as features in a classifier algorithm [38-41]. Through our simple procedure, common CNV signatures can be identified that can be readily applied to other datasets with similar data types as demonstrated with our use of a test set in this report. These advantages along with our reported and validated caCNV signature gives credence to our novel approach which could also easily be implemented to identify CNVs as susceptibility loci in case–control studies.

Admixture in the test set is a possible limitation of our study. Admixture was expected in African-American cohort, and the probabilities of identifying African ancestry in the test set was lower than that obtained in our training set. Nonetheless, we showed high correlation between the estimated posterior probability of ancestry from the caCNV signature to estimates of admixture from genome-wide SNP data using ADMIXTURE software.

Application of common CNVs can complement informative SNPs in ancestral studies or case–control studies. Common CNVs may encompass genes giving rise to the observed phenotype, and do not necessarily rely on linkage disequilibrium with the underlying causal variant. The likelihood of gene dosage effects of CNVs can also provide insight to the biological differences observed between populations. Finally, future studies could explore the combination of CNVs and SNPs to identify population stratification.

## Conclusions

In summary, we described a new methodology to identify common CNVs and demonstrated its performance by building a caCNV signature to predict human ancestry. Our novel approach reveals a 73 caCNV signature, which 73% of the caCNVs have been confirmed by other approaches and can be used to infer human population structure with extremely high accuracy. A simple modification of the GADA method allowed for direct segmentation of t-statistics to identify the caCNVs. The efficiency of our method in finding CNV signatures will facilitate the use of a new type of structural variation important in human genomic studies. The success of our methodology has implications for improving admixture mapping and the minimization of population stratification in case–control and genome-wide association studies. This methodology can be easily expanded to large studies aimed to identify a genetic susceptibility CNV signature specific to other phenotypes such as disease or drug response.

## Abbreviations

CNV: Copy number variation; SNP: Single nucleotide polymorphism; GADA: Genome alteration detection analysis; caCNV: Common ancestry copy number variation; aCGH: Array-based comparative genome hybridization; SBL: Sparse bayesian learning; BE: Backward elimination; Affy SNP 6.0: Affymetrix Genome-Wide Human SNP Array 6.0; CEU: HapMap individuals of European descent from Utah; YRI: HapMap individuals of the African Yoruba from Nigeria; CHB: Unrelated Han Chinese HapMap individuals from Beijing China; CGEMS: Cancer Genetic Markers of Susceptibility; NIGMS: National Institute of General Medical Sciences; LDA: Linear discriminant analysis; PCA: Principal component analysis; AIMs: Ancestry informative markers.

## Competing interests

The authors declare that they have no competing interests.

## Authors’ contributions

MW carried out the statistical analysis and drafted the manuscript. RP provided critical comments and suggestions. MV performed essential data analysis. SA conceptualized the methodology, participated in the design and coordination of the study, and helped to draft the manuscript. All authors read and approved the final manuscript.

## Supplementary Material

Additional file 1 Table S1. CNVs identified in individual samples. Click here for file

Additional file 2 Table S2.Detailed list of the 73 caCNV signature. Click here for file

Additional file 3 Figure S1. Accuracy of Ancestry Prediction in Test Set using PCA of Genome-Wide SNPs. **A**) Scatter plot of the top two principal components using data generated from 4,326 genome-wide SNPs selected as ancestry informative markers (AIMs) shows separation of 100 European, 100 African-American, and 100 Han Chinese test samples based on self-reported ancestry (red square: European; yellow triangle: African-American; blue circle: Han Chinese). **B**) Scatter plot of ancestry estimates using SNPs versus caCNV signature in Africans (R^2^ = 0.914), **C**) Europeans (R^2^ = 0.924), and **D**) Han Chinese (R^2^ = 0.974). Click here for file
