# Supplementary figures and images for: Copy number variation signature to predict human ancestry
Source: BMC Bioinformatics. 2012 Dec 27;13:336. doi: 10.1186/1471-2105-13-336 (PMC3598683; doi:10.1186/1471-2105-13-336)

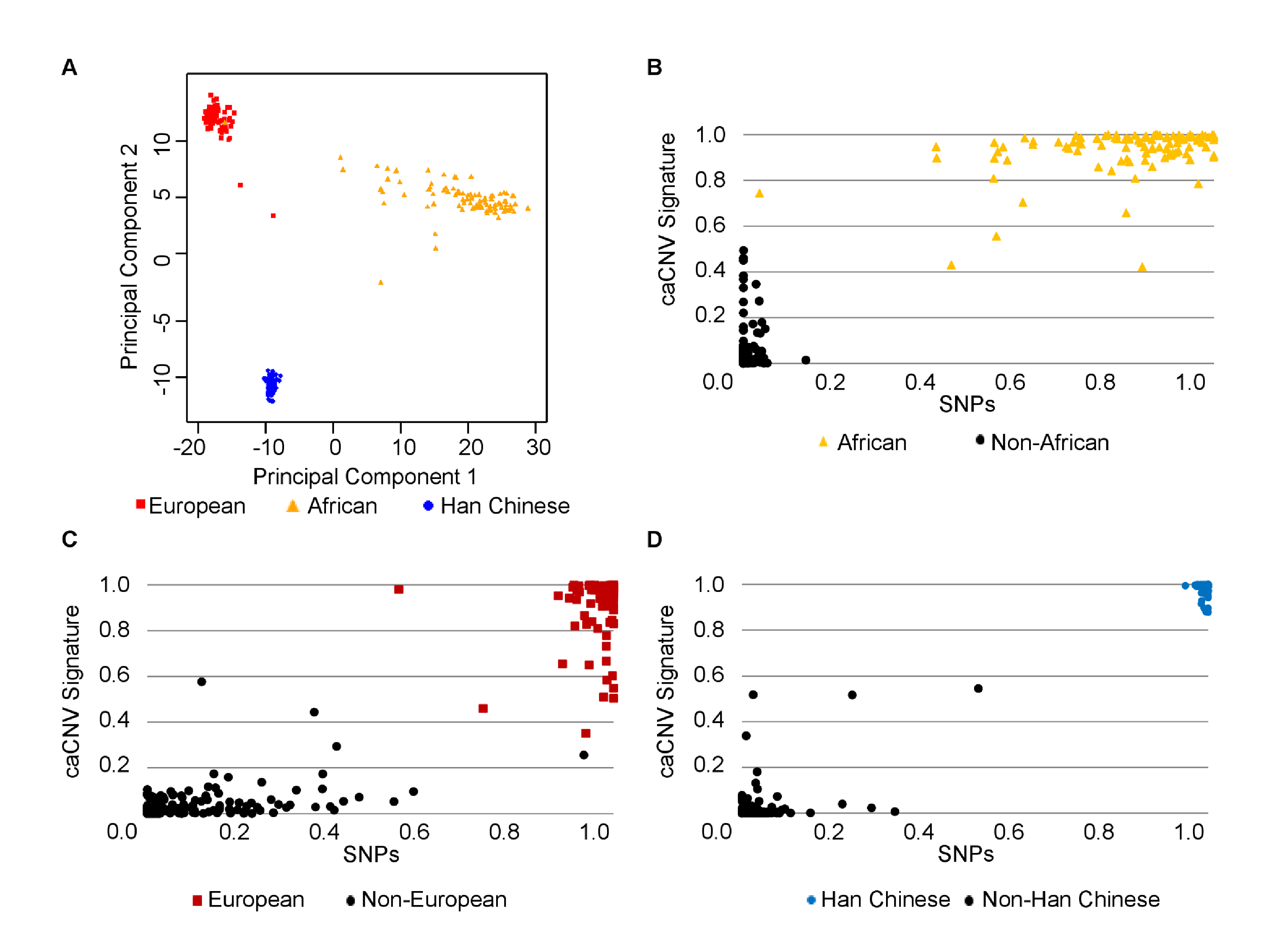

Supplement: Additional file 3 — Figure S1. Accuracy of Ancestry Prediction in Test Set using PCA of Genome-Wide SNPs. A) Scatter plot of the top two principal components using data generated from 4,326 genome-wide SNPs selected as ancestry informative markers (AIMs) shows separation of 100 European, 100 African-American, and 100 Han Chinese test samples based on self-reported ancestry (red square: European; yellow triangle: African-American; blue circle: Han Chinese). B) Scatter plot of ancestry estimates using SNPs versus caCNV signature in Africans (R2 = 0.914), C) Europeans (R2 = 0.924), and D) Han Chinese (R2 = 0.974). [file 1471-2105-13-336-S3.tiff]
